# (2,4-Dihydroxy­benzyl­idene)dimethyl­ammonium dichloro­phosphinate

**DOI:** 10.1107/S1600536809001925

**Published:** 2009-01-23

**Authors:** Hai-Jun Xu, Qing-Yin Tan, Yi-Jie Pang

**Affiliations:** a Ordered Matter Science Research Center, College of Chemistry and Chemical, Engineering, Southeast University, Nanjing 210096, People’s Republic of China

## Abstract

In the title compound, C_9_H_12_NO_2_^+^·Cl_2_PO_2_^−^, the mol­ecular skeleton of the cation is nearly planar with an r.m.s. deviation of 0.0336 Å. In the crystal structure, inter­molecular O—H⋯O hydrogen bonds link cations and anions into chains running along [1
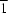
0].

## Related literature

For details of the synthesis, see Ramadas & David Krupadanam (2000 ▶). For typical values of C=N bond lengths, see Elmah *et al.* (1999 ▶).
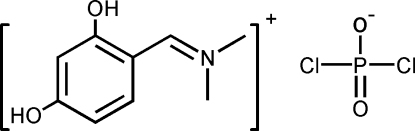


## Experimental

### 

#### Crystal data

C_9_H_12_NO_2_^+^·Cl_2_O_2_P^−^
*M* *_r_* = 300.07
Triclinic, 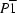

*a* = 7.922 (4) Å
*b* = 8.163 (4) Å
*c* = 11.035 (9) Å
α = 100.021 (19)°
β = 107.035 (2)°
γ = 103.02 (3)°
*V* = 642.3 (7) Å^3^
*Z* = 2
Mo *K*α radiation
μ = 0.63 mm^−1^
*T* = 293 (2) K
0.20 × 0.20 × 0.20 mm

#### Data collection

Rigaku Mercury2 diffractometer
Absorption correction: multi-scan (*CrystalClear*; Rigaku, 2005 ▶) *T*_min_ = 0.903, *T*_max_ = 1.000 (expected range = 0.796–0.881)
6473 measured reflections
2898 independent reflections
2374 reflections with *I* > 2σ(*I*)
*R*_int_ = 0.020

#### Refinement

*R*[*F* ^2^ > 2σ(*F* ^2^)] = 0.043
*wR*(*F* ^2^) = 0.116
*S* = 1.06
2898 reflections
155 parameters
H-atom parameters constrained
Δρ_max_ = 0.34 e Å^−3^
Δρ_min_ = −0.38 e Å^−3^

### 

Data collection: *CrystalClear* (Rigaku, 2005 ▶); cell refinement: *CrystalClear*; data reduction: *CrystalClear*; program(s) used to solve structure: *SHELXS97* (Sheldrick, 2008 ▶); program(s) used to refine structure: *SHELXL97* (Sheldrick, 2008 ▶); molecular graphics: *SHELXTL* (Sheldrick, 2008 ▶); software used to prepare material for publication: *SHELXL97*.

## Supplementary Material

Crystal structure: contains datablocks I, global. DOI: 10.1107/S1600536809001925/cv2507sup1.cif

Structure factors: contains datablocks I. DOI: 10.1107/S1600536809001925/cv2507Isup2.hkl

Additional supplementary materials:  crystallographic information; 3D view; checkCIF report

## Figures and Tables

**Table 1:** Hydrogen-bond geometry (Å, °)

*D*—H⋯*A*	*D*—H	H⋯*A*	*D*⋯*A*	*D*—H⋯*A*
O1—H1*A*⋯O4^i^	0.82	1.79	2.609 (3)	180
O2—H2⋯O3^ii^	0.82	1.83	2.635 (3)	167

Symmetry codes: (i) 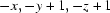; (ii) 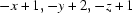.

**Fig. 1.**
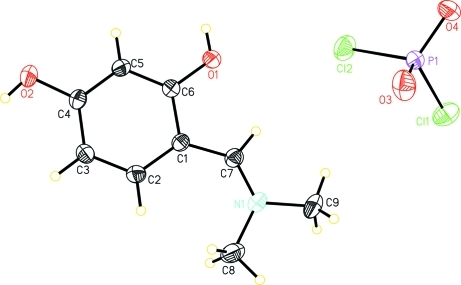
A view of the title compound with the atomic numbering scheme. Displacement ellipsoids are drawn at the 30% probability level.

## supplementary crystallographic information

### Comment

Vilsmeier conditions are important synthetic tool usually utilized in the synthesis of aldehydes . The title compound is a Vilsmeier intermediate synthesized from resorcinol, DMF and POCl_3_ in dry CH_3_CN. Here we report its crystal structure.

In the title compound (Fig. 1), all bond lengths are normal. The C7=N1 bond length of 1.288 (3) Å indicates a high degree of double-bond character comparable with the typical values of C=N bond length (Elmah *et al.*, 1999). The two P—O bond lengths are almost equal - 1.4604 (19)  and 1.4642 (18) Å, repectively.

In the crystal, the cations and anions are further connected via O–H···O hydrongen bonds into chains running in direction [1-10].

### Experimental

All chemicals were obtained from commercial sources and used without further purification except POCl_3_ and DMF, which were distiled under reduced pressure before use. The title compound was prepared according to the literature (Ramadas & David Krupadanam, 2000).

### Refinement

All H atoms were geometrically positioned (C—H 0.93-0.96 Å, O—H 0.82 Å) and allowed to ride on the parent atoms, with *U*_iso_(H) = 1.2-1.5 *U*_eq_(C, O).

### Crystal data

**Table:**

C_9_H_12_NO_2_^+^·Cl_2_O_2_P^−^	*Z* = 2
*M**_r_* = 300.07	*F*(000) = 308
Triclinic, *P*1	*D*_x_ = 1.552 Mg m^−^^3^
Hall symbol: -P 1	Mo *K*α radiation, λ = 0.71073 Å
*a* = 7.922 (4) Å	Cell parameters from 1854 reflections
*b* = 8.163 (4) Å	θ = 2.7–27.5°
*c* = 11.035 (9) Å	µ = 0.63 mm^−^^1^
α = 100.021 (19)°	*T* = 293 K
β = 107.035 (2)°	Prism, colourless
γ = 103.02 (3)°	0.20 × 0.20 × 0.20 mm
*V* = 642.3 (7) Å^3^	

### Data collection

**Table:**

Rigaku Mercury2 diffractometer	2898 independent reflections
Radiation source: fine-focus sealed tube	2374 reflections with *I* > 2σ(*I*)
graphite	*R*_int_ = 0.020
Detector resolution: 13.6612 pixels mm^-1^	θ_max_ = 27.5°, θ_min_ = 2.7°
ω scans	*h* = −10→10
Absorption correction: multi-scan (*CrystalClear*; Rigaku, 2005)	*k* = −10→10
*T*_min_ = 0.903, *T*_max_ = 1.000	*l* = −14→14
6473 measured reflections	

### Refinement

**Table:**

Refinement on *F*^2^	Primary atom site location: structure-invariant direct methods
Least-squares matrix: full	Secondary atom site location: difference Fourier map
*R*[*F*^2^ > 2σ(*F*^2^)] = 0.043	Hydrogen site location: inferred from neighbouring sites
*wR*(*F*^2^) = 0.116	H-atom parameters constrained
*S* = 1.06	*w* = 1/[σ^2^(*F*_o_^2^) + (0.0563*P*)^2^ + 0.2201*P*] where *P* = (*F*_o_^2^ + 2*F*_c_^2^)/3
2898 reflections	(Δ/σ)_max_ < 0.001
155 parameters	Δρ_max_ = 0.34 e Å^−^^3^
0 restraints	Δρ_min_ = −0.38 e Å^−^^3^

### Special details

**Table:**

Geometry. All e.s.d.'s (except the e.s.d. in the dihedral angle between two l.s. planes) are estimated using the full covariance matrix. The cell e.s.d.'s are taken into account individually in the estimation of e.s.d.'s in distances, angles and torsion angles; correlations between e.s.d.'s in cell parameters are only used when they are defined by crystal symmetry. An approximate (isotropic) treatment of cell e.s.d.'s is used for estimating e.s.d.'s involving l.s. planes.
Refinement. Refinement of *F*^2^ against ALL reflections. The weighted *R*-factor *wR* and goodness of fit *S* are based on *F*^2^, conventional *R*-factors *R* are based on *F*, with *F* set to zero for negative *F*^2^. The threshold expression of *F*^2^ > σ(*F*^2^) is used only for calculating *R*-factors(gt) *etc*. and is not relevant to the choice of reflections for refinement. *R*-factors based on *F*^2^ are statistically about twice as large as those based on *F*, and *R*- factors based on ALL data will be even larger.

### Fractional atomic coordinates and isotropic or equivalent isotropic displacement parameters (Å^2^)

**Table:**

	*x*	*y*	*z*	*U*_iso_*/*U*_eq_	
P1	0.07808 (7)	0.57775 (7)	0.79425 (5)	0.04024 (16)	
Cl2	−0.01734 (10)	0.66505 (10)	0.63397 (6)	0.0665 (2)	
O1	0.1067 (2)	0.88448 (19)	0.41735 (15)	0.0469 (4)	
H1A	0.0823	0.7961	0.3592	0.070*	
O2	0.4455 (2)	1.1302 (2)	0.17313 (15)	0.0556 (4)	
H2	0.5384	1.2072	0.1825	0.083*	
C1	0.3561 (2)	1.1426 (2)	0.52665 (18)	0.0340 (4)	
N1	0.3863 (2)	1.2128 (2)	0.76201 (16)	0.0424 (4)	
C6	0.2445 (3)	1.0094 (2)	0.40992 (19)	0.0342 (4)	
C5	0.2758 (3)	1.0099 (3)	0.29337 (19)	0.0382 (4)	
H5A	0.2007	0.9231	0.2179	0.046*	
C7	0.3111 (3)	1.1237 (3)	0.6409 (2)	0.0394 (4)	
H7A	0.2087	1.0299	0.6251	0.047*	
C3	0.5312 (3)	1.2722 (3)	0.4014 (2)	0.0424 (5)	
H3A	0.6267	1.3596	0.3980	0.051*	
C4	0.4190 (3)	1.1396 (3)	0.28853 (19)	0.0388 (4)	
C2	0.4993 (3)	1.2723 (3)	0.5167 (2)	0.0411 (5)	
H2A	0.5746	1.3609	0.5911	0.049*	
C8	0.5515 (4)	1.3646 (4)	0.8156 (2)	0.0627 (7)	
H8A	0.5790	1.4070	0.9081	0.094*	
H8B	0.5293	1.4545	0.7729	0.094*	
H8C	0.6541	1.3315	0.8009	0.094*	
C9	0.3073 (4)	1.1623 (4)	0.8600 (2)	0.0642 (7)	
H9A	0.2007	1.0624	0.8178	0.096*	
H9B	0.2720	1.2571	0.8998	0.096*	
H9C	0.3977	1.1345	0.9261	0.096*	
Cl1	0.00162 (13)	0.72157 (11)	0.92482 (8)	0.0820 (3)	
O4	−0.0294 (3)	0.3961 (2)	0.76791 (18)	0.0643 (5)	
O3	0.2793 (2)	0.6276 (3)	0.83673 (19)	0.0676 (5)	

### Atomic displacement parameters (Å^2^)

**Table:**

	*U*^11^	*U*^22^	*U*^33^	*U*^12^	*U*^13^	*U*^23^
P1	0.0420 (3)	0.0381 (3)	0.0372 (3)	0.0040 (2)	0.0153 (2)	0.0083 (2)
Cl2	0.0797 (5)	0.0756 (5)	0.0469 (4)	0.0299 (4)	0.0158 (3)	0.0234 (3)
O1	0.0444 (8)	0.0444 (8)	0.0439 (8)	−0.0018 (6)	0.0192 (7)	0.0040 (6)
O2	0.0622 (11)	0.0637 (11)	0.0367 (8)	0.0029 (8)	0.0235 (7)	0.0116 (7)
C1	0.0336 (9)	0.0374 (10)	0.0300 (9)	0.0116 (8)	0.0087 (7)	0.0087 (7)
N1	0.0439 (10)	0.0527 (11)	0.0315 (9)	0.0174 (8)	0.0129 (7)	0.0091 (7)
C6	0.0322 (9)	0.0349 (9)	0.0357 (10)	0.0108 (7)	0.0110 (7)	0.0096 (7)
C5	0.0402 (10)	0.0382 (10)	0.0320 (10)	0.0094 (8)	0.0104 (8)	0.0047 (8)
C7	0.0376 (10)	0.0439 (11)	0.0362 (10)	0.0121 (8)	0.0121 (8)	0.0098 (8)
C3	0.0410 (11)	0.0408 (11)	0.0406 (11)	0.0031 (8)	0.0132 (9)	0.0121 (9)
C4	0.0427 (11)	0.0439 (11)	0.0323 (10)	0.0138 (8)	0.0137 (8)	0.0133 (8)
C2	0.0402 (11)	0.0395 (10)	0.0349 (10)	0.0050 (8)	0.0074 (8)	0.0067 (8)
C8	0.0578 (15)	0.0714 (17)	0.0398 (13)	0.0026 (12)	0.0120 (11)	−0.0034 (11)
C9	0.0794 (18)	0.0802 (18)	0.0377 (12)	0.0198 (14)	0.0290 (12)	0.0162 (12)
Cl1	0.1114 (7)	0.0773 (5)	0.0594 (4)	0.0271 (4)	0.0444 (4)	−0.0020 (3)
O4	0.0824 (13)	0.0392 (9)	0.0644 (12)	−0.0028 (8)	0.0353 (10)	0.0061 (8)
O3	0.0420 (9)	0.0902 (14)	0.0699 (12)	0.0091 (9)	0.0166 (8)	0.0371 (10)

### Geometric parameters (Å, °)

**Table:**

P1—O3	1.4598 (19)	C6—C5	1.380 (3)
P1—O4	1.4641 (18)	C5—C4	1.387 (3)
P1—Cl1	2.0160 (13)	C5—H5A	0.9300
P1—Cl2	2.0262 (15)	C7—H7A	0.9300
O1—C6	1.349 (2)	C3—C2	1.367 (3)
O1—H1A	0.8200	C3—C4	1.401 (3)
O2—C4	1.342 (3)	C3—H3A	0.9300
O2—H2	0.8200	C2—H2A	0.9300
C1—C2	1.412 (3)	C8—H8A	0.9600
C1—C6	1.426 (3)	C8—H8B	0.9600
C1—C7	1.431 (3)	C8—H8C	0.9600
N1—C7	1.292 (3)	C9—H9A	0.9600
N1—C8	1.470 (3)	C9—H9B	0.9600
N1—C9	1.471 (3)	C9—H9C	0.9600
			
O3—P1—O4	120.75 (12)	C1—C7—H7A	114.0
O3—P1—Cl1	109.16 (10)	C2—C3—C4	119.5 (2)
O4—P1—Cl1	107.38 (8)	C2—C3—H3A	120.2
O3—P1—Cl2	108.40 (8)	C4—C3—H3A	120.2
O4—P1—Cl2	108.48 (9)	O2—C4—C5	117.32 (18)
Cl1—P1—Cl2	100.85 (7)	O2—C4—C3	122.41 (19)
C6—O1—H1A	109.5	C5—C4—C3	120.26 (19)
C4—O2—H2	109.5	C3—C2—C1	122.27 (18)
C2—C1—C6	116.78 (18)	C3—C2—H2A	118.9
C2—C1—C7	128.16 (18)	C1—C2—H2A	118.9
C6—C1—C7	115.03 (18)	N1—C8—H8A	109.5
C7—N1—C8	125.83 (19)	N1—C8—H8B	109.5
C7—N1—C9	119.8 (2)	H8A—C8—H8B	109.5
C8—N1—C9	114.33 (19)	N1—C8—H8C	109.5
O1—C6—C5	121.34 (17)	H8A—C8—H8C	109.5
O1—C6—C1	117.69 (17)	H8B—C8—H8C	109.5
C5—C6—C1	120.97 (18)	N1—C9—H9A	109.5
C6—C5—C4	120.18 (18)	N1—C9—H9B	109.5
C6—C5—H5A	119.9	H9A—C9—H9B	109.5
C4—C5—H5A	119.9	N1—C9—H9C	109.5
N1—C7—C1	131.9 (2)	H9A—C9—H9C	109.5
N1—C7—H7A	114.0	H9B—C9—H9C	109.5

### Hydrogen-bond geometry (Å, °)

**Table:**

*D*—H···*A*	*D*—H	H···*A*	*D*···*A*	*D*—H···*A*
O1—H1A···O4^i^	0.82	1.79	2.609 (3)	180
O2—H2···O3^ii^	0.82	1.83	2.635 (3)	167

Symmetry codes: (i) −*x*, −*y*+1, −*z*+1; (ii) −*x*+1, −*y*+2, −*z*+1.
